# Edaphic specialization onto bare, rocky outcrops as a factor in the evolution of desert angiosperms

**DOI:** 10.1073/pnas.2214729120

**Published:** 2023-01-30

**Authors:** Isaac H. Lichter-Marck, Bruce G. Baldwin

**Affiliations:** ^a^Department of Integrative Biology and Jepson Herbarium, University of California, Berkeley, CA 94720; ^b^Department of Ecology and Evolutionary Biology, University of California, Los Angeles, CA 90095

**Keywords:** adaptation, biome-shifts, deserts, edaphic specialization, pre-adaptation

## Abstract

Understanding the processes that enable organisms to shift into more arid environments as they emerge is critical for gauging resilience to climate change, yet these forces remain poorly known. In a comprehensive clade-based study, we investigate recent shifts into North American deserts in the rock daisies (tribe Perityleae), a diverse tribe of desert sunflowers (Compositae). We sample rock daisies across two separate contact zones between tropical deciduous forest and desert biomes in western North America and infer a time-calibrated phylogeny based on target capture sequence data. We infer biome shifts using Bayesian inference with paleobiome-informed models and find evidence for seven independent shifts into desert habitats since the onset of aridification in the late Miocene. The earliest shift occurred out of tropical deciduous forests and led to an extensive radiation throughout North American deserts that accounts for the majority of extant desert rock daisies. Estimates of life history and micro-habitat in the rock daisies reveal a correlation between a suffrutescent perennial life history and edaphic endemism onto rocky outcrops, an ecological specialization that evolved prior to establishment and diversification in deserts. That the insular radiation of desert rock daisies stemmed from ancestors preadapted for dry conditions as edaphic endemics in otherwise densely vegetated tropical deciduous forests in northwest Mexico underscores the crucial role of exaptation and dispersal for shifts into arid environments.

## Significance

The environmentally stressful conditions found in desert regions have often been implicated as the main factor in the evolution of drought tolerance in desert plants. Yet, many iconic desert plant lineages evolved prior to the recent emergence of widespread arid climates, suggesting an important role for preadaptation (exaptation). We provide empirical support for this view by showing that life-history evolution associated with ecological specialization onto rock outcrops was a precursor to establishment and extensive diversification in North American deserts in the desert rock daisies (Perityleae). We caution against assuming the presence of ancient dry biomes based on time-calibrated phylogenies and we emphasize the potentially limited responses of organisms to increasing aridity caused by global climate change.

Desert biomes cover nearly a fifth of earth’s surface and are predicted to expand as a result of climate change (1, 2). Yet, we still have much to learn about the potential for biotic communities to adapt to new levels of increased aridity. Compared with other biomes, many deserts emerged recently on a geological timescale (3); therefore, their endemic biota provides an ideal system for understanding the organismal potential to fill novel environments created by future climate change. North American (NA) deserts cover most of the southwest U.S. and northern Mexico (4) and appeared for the first time in scattered, ephemeral pockets as recently as the late-Miocene during Neogene uplift (5 to 7 Ma) and then expanded during the Pliocene (2.4 to 5 Ma) (5). Despite their recent origin, NA deserts contain a diverse and endemic flora and fauna endowed with striking abilities to escape or tolerate their harsh, arid climate whose origins have been a topic of debate among evolutionary biologists for decades (6, 7). Examples of evolutionary adaptation showcased by desert plants include the ephemeral annual lifestyle, and the many types of unique, stunted, thorny, or succulent vegetative growth forms that are features well-suited for drought tolerance or escape (4, 8). Most commonly, these features are attributed to rapid adaptive evolution in response to the environmentally stressful conditions that characterize deserts: unpredictable precipitation, extremes of temperature, and coarse, rocky soils (9). Yet, this explanation has been difficult to reconcile with the recent emergence of deserts and the age of origin of iconic desert plant lineages in the Oligocene supported by time-calibrated phylogenies and the fossil record (10–12). An alternative explanation is that desert plants evolved by exaptation in arid microsites, such as rain-shadows or rock outcrops, within the moist forests of older biomes, and then spread and diversified when the climate became cooler and drier (7). Thus, transitions into desert biomes may have been accomplished by rapid adaptive evolution, or organisms preadapted to novel conditions within neighboring biomes may have dispersed into arid environments as they emerged (13).

While the phylogenetic and statistical tools needed to tease apart the roles of adaptive evolution and dispersal during biome shifts have grown substantially in recent years, testing these alternative hypotheses continues to pose a challenge because of the difficulty in pinpointing when and where evolutionary events of particular importance happened from contemporary data (14). Phylogenetic studies set within a paleobiome and paleogeographically informed framework have the potential to resolve when and where biome shifts have occurred in the past (15). Yet, rapid adaptation or exaptation, by definition, implies both an evolutionary transition in form as well as a shift in ecological context. Only comprehensive clade-based studies integrating both extrinsic (environmental) and intrinsic (trait) data into a time informed context can provide the varied lines of evidence needed to evaluate hypotheses about the mechanisms underlying biome shifts (14). Here, we investigate biome shifts into NA deserts using a uniquely comprehensive study of rock-daisies (tribe Perityleae) of the sunflower family (Compositae). We infer shifts into desert biomes using Bayesian inference with paleobiome-informed models, then draw upon unique life-history traits closely tied to microclimate specificity to build an integrative understanding of ecological transitions and trait evolution through time for 73 out of 84 species of rock daisies.

**Fig. 1.**
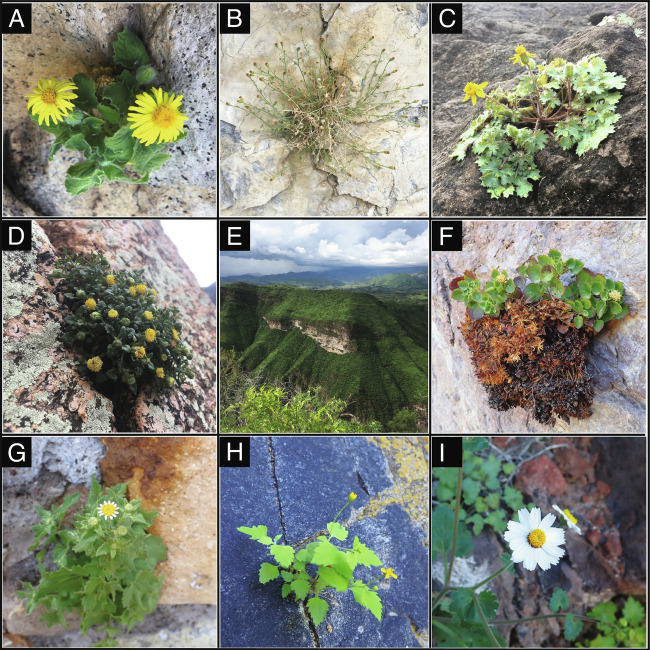
Habit and habitat diversity of rock daisies (Perityleae). (*A*) *Laphamia cordifolia*; (*B*) *Laphamia villosa;* (*C*) *Laphamia lobata;* (*D*) *Laphamia cochisensis*; (*E*) tropical deciduous forest with bare, rocky outcrops in the Sierra Madre Occidental in Sonora, Mexico; (*F*) *Laphamia cernua;* (*G*) *Galinsogeopsis spilanthoides*; (*H*) *Perityle cuneata* var. *cuneata;* (*I*) *Perityle rotundifolia;* Photos by ILM.

The evolution of the megadiverse sunflower family is closely tied to the emergence of arid biomes worldwide, but compared with other dryland plant groups, such as legumes, cacti, or grasses, few studies have examined ecological transitions to arid climates in desert Compositae (10, 16–18). Rock daisies are one of the most diverse groups of composites found in the NA deserts, and their range extends to tropical deciduous forests adjacent to desert in northwest mainland Mexico and the Baja California peninsula (19, 20). Disjunct populations are also present in the Atacama Desert in Chile (19, 20). Within the NA deserts, rock daisies show characteristics suggestive of an insular radiation, including high species diversity, geographic speciation, divergent life-history strategies, and specialization into disparate ecological niches (20, 21). A remarkable aspect of this group’s natural history is the extreme edaphic specialization demonstrated by some taxa, which grow exclusively on bare, rocky outcrops and cliffs on the slopes of desert mountains. Rock daisies associate with bare rock of diverse types (lithologies) in both desert and tropical habitats. Growth on solid rock may be facilitated by a specialized life-history trait, a perennating woody caudex lodged into cracks or crevices from which herbaceous shoots resprout annually (suffrutescent perennial life history, Fig. 1). Other rock daisies lack this trait and these may be multibranched woody shrubs, herbaceous perennials, or annuals, including some of the most abundant ephemeral wildflowers (e.g., *Perityle emoryi* Torr., *Perityle californica* Benth., *Galinsogeopsis spilanthoides* Sch. Bip.) found in the seasonal blooms of low deserts in favorable years (19, 20). As in other groups of Compositae, rock daisies show biological attributes conducive to the colonization of stressful niches, including habitual breakdown of reproductive self-incompatibility, a generalized pollination strategy, diverse secondary metabolite chemistry, glandular foliage, and rapid pappus (calyx) evolution, which serves a dual function for dispersal to isolated habitats and defense against seed predation (19, 21). Rock daisies are also rare; with >30% of species listed as vulnerable, imperiled, or critically endangered according to NatureServe (22), they have one of the highest proportions of at-risk taxa of any group of desert plants in western North America.

The ecogeographic range, breadth of ecological specializations, and life-history variation exhibited by rock daisies makes them an ideal group to study the roles of adaptive evolution and dispersal on biome shifts into the NA deserts; however, a densely sampled phylogeny of this lineage has only recently become available (21). A robust phylogeny for rock daisies based on target capture using the Conserved Compositae Ortholog set (COS) (21) formed the basis for recent reclassification of the group into 9 genera (20). Phylogenies based on target-capture data have proved useful in resolving relationships in this group at a variety of scales, but more sampling has been needed to fill gaps in previous phylogenies. Here, we describe the results of the most densely sampled phylogenetic study of rock daisies to date based on target capture of 73 out of 84 recognized species. We employ our improved resolution of relationships to test hypotheses regarding shifts into desert biomes and onto harsh edaphic substrates, with a focus on the texture and structure axis of edaphic environments, rather than chemical properties. Specifically, we test whether transitions to key functional traits for enduring arid conditions occurred as an adaptive response to the environmentally stressful barriers presented by desert habitats, or whether rock daisies that were preadapted (exapted) to arid conditions on edaphically bare microsites within older biomes dispersed into deserts with corresponding trait evolution already in place.

## Results and Discussion

### Insights into the Evolutionary and Biogeographic History of the Rock Daisies.

Does phylogenomic-scale data resolve relationships among the rock daisies? Phylogenies of rock daisies based on a concatenated data matrix of 1,419,391 bp of orthologous nuclear loci were highly resolved at most nodes for both maximum likelihood (ML) and Bayesian inference (BI) approaches and congruent with each other, as well as with previously published phylogenies of rock daisies based on COS loci (*SI Appendix*, Figs. S2, S5, and S6; 21). Sampling of almost all recognized taxa allowed for the most comprehensive understanding of evolutionary relationships among rock daisies yet (*SI Appendix*, Fig. S2). Conspecific samples clustered together for most taxa, but many were found to be nonmonophyletic, suggesting the presence of multiple cryptic lineages in need of taxonomic follow-up. Lack of resolution within individual loci or discordance due to ILS and introgressive hybridization was in evidence in our pseudocoalescent phylogenetic analysis, with a major topological difference between the pseudocoalescent tree and the concatenated analysis being the placement of the widespread allopolyploid *P. emoryi,* which resolves as nonmonophyletic and sister (in part) to *Laphamia* in our pseudocoalescent tree but as nested in *Perityle* with high support in all other analyses (*SI Appendix*, Figs. S5–S7). Nonmonophyletic taxa grouped together within biomes, edaphic habitats, and life-history types, such that cryptic diversity was unlikely to bias character coding or influence transition rates presented in this study. However, in producing a reduced sample for divergence-time estimation that included only one terminal per taxon, individuals were retained from each independent evolutionary lineage when taxa were found to be nonmonophyletic.

**Fig. 2.**
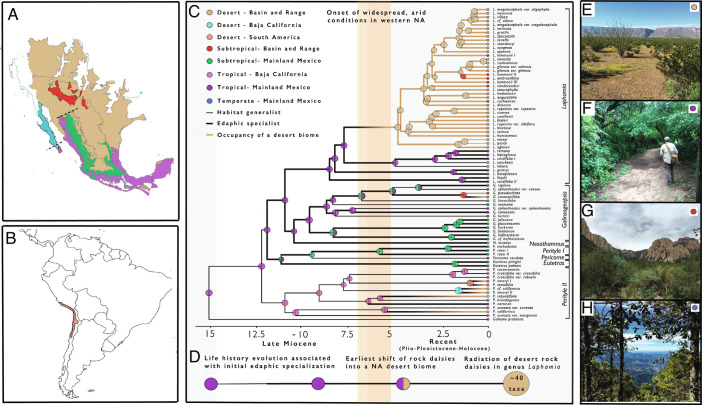
Establishment and diversification of the rock daisies in the North American desert biome (*A*) Biome map of North America based on level I ecoregions published by the Intergovernmental Commission for Environmental Cooperation illustrating the biome-area combinations addressed in this study. Colors indicate desert in the Basin and Range Province (brown); desert in Baja California (blue); subtropical forest in the Basin and Range Province (red); subtropical forest in the Sierra Madre Occidental and Trans-Mexican Volcanic Belt of Mexico (“mainland Mexico,” green); frost-free, tropical deciduous forest in Baja California (pink); and tropical habitats in mainland Mexico (purple). As defined here, biomes include a variety of minor, yet distinctive vegetation types, such as coastal plain, mangrove, and rocky sites. Dashed lines indicate two geographically separated contact zones between tropical deciduous forest and desert biomes in mainland Mexico and Baja California Sur. (*B*) Distribution of the geographically disjunct Atacama Desert (orange) in South America. (*C*) Maximum a posteriori (MAP) estimates of ancestral biome-area combinations in the rock daisies inferred using the biome-shift model of Landis et al. (8). MAP estimates were compiled from 23,500 post-burn-in iterations of MCMC in RevBayes (26). Colors at nodes indicate biome-region combinations corresponding to colors in (*A*), (*B*), and the legend provided. Ancestral states with posterior probabilities less than 0.9 are illustrated as split circles in combination with the second most probable biome-area on the right. Occupation of a desert biome is indicated by brown branches and biome-shifts into deserts are shown as branches with a gradient of coloration. Ecological specialization onto bare, rocky outcrops is represented by thick branches, while habitat generalism is represented with thin branches. Shaded background bars separate the time intervals used in our model of paleobiome and paleogeographic structure. The vertical orange bar indicates the timing of emergence of widespread arid habitats in western North America. (*D*) A timeline gives the relative estimates of timing of initial life-history evolution associated with ecological specialization, biome-shifts into deserts, and radiation of desert rock daisies in the genus *Laphamia.* (*E*–*H*) Landscape photographs of dominant vegetation types in biomes included in this study. (*E*) Desert plain in Big Bend National Park in Texas, U.S.A. (*F*) Tropical deciduous forest in the Sierra de Alamos in Sonora, Mexico. (*G*) Subtropical woodland in the Chiricahua Mountains of Arizona, U.S.A. (*H*) Temperate forest in the Sierra Madre Occidental of Durango, Mexico. Photos by ILM.

**Fig. 3.**
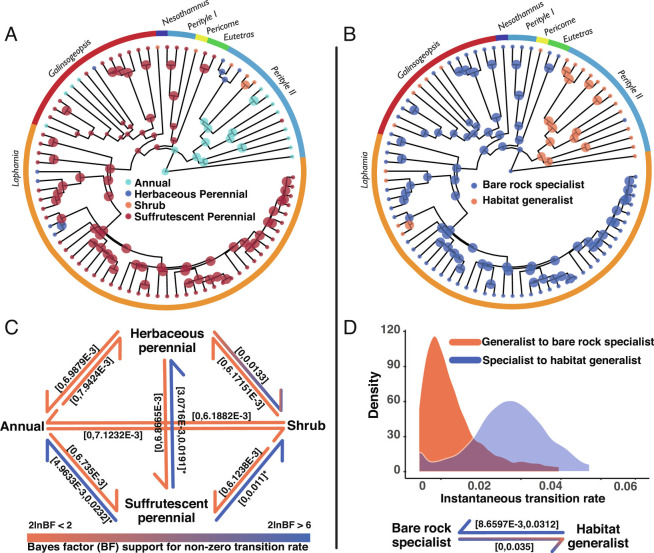
Evolution of edaphic specialization onto bare, rocky outcrops is closely correlated with a suffrutescent life history and evolved early in the evolutionary history of rock daisies. (*A*) Model-averaged maximum a posteriori (MAP) ancestral state estimate of the woody shrub, suffrutescent perennial, herbaceous perennial, and annual life histories based on 13,500 iterations of post-burn-in rjMCMC in RevBayes (26); (*B*) MAP ancestral state estimate of strict edaphic endemism on bare rock and habitat generalism in the rock daisies based on 13,500 iterations of post-burn-in rjMCMC in RevBayes (26); (*C*) Inferred transition rates (95% HPD intervals) between life histories. A blue color indicates strong support for a nonzero transition rate (2log Bayes factor > 6), while an orange color indicates equivocal support for a transition rate being significantly different from zero (2log BF < 2). (*D*) Posterior density estimates of transition rate asymmetries between obligate growth on bare rock (bare rock specialism) and occurrence on decomposed substrates (habitat generalism). Inferred transition rates (95% HPD intervals) are color coded following the key provided in (*C*).

Do estimates of the timing of rock daisy evolution show concordance with major paleogeographical and paleoecological events? In our reduced sample, maximum-clade-credibility time-calibrated phylogeny, the root node corresponding to the most recent common ancestor of tribes Perityleae+Eupatorieae and *Helianthus annuus* L. was inferred to have a median age of 15.8033 Ma [9.6371 to 20.8277 Ma, 95% HPD] and agrees with the range of times found in previous dating analysis of the Heliantheae alliance (23, 24, *SI Appendix*, Fig. S3*A*). The split between the two major lineages of rock daisies is inferred at 12.5643 Ma [7.7409 to 17.1751 Ma, 95% HPD] and shows remarkable correspondence in timing with estimates of splitting of the Baja California peninsula from mainland Mexico (25,  *SI Appendix*, Fig. S3*B*). Indeed, a cladogenetic (subset sympatry) event resolved at this node separates the principal radiations of rock daisies, which have distributions centered on mainland Mexico and Baja California, respectively (*SI Appendix*, Fig. S4*A*). Recent paleobiome studies based on fossil, pollen, and imputed climatological data infer a range of first emergence of widespread desert habitats in western NA ~5 to 7 Ma, with climatic cooling and aridification leading to further spread of arid environments during the Pliocene (5). For the most part, divergence events separating the major clades of rock daisies predate widespread climatic aridification and occur prior to dispersal into the Basin and Range Province within more densely vegetated areas of northwest Mexico (Figs. 2 and 3 and *SI Appendix*, Figs. S3*C* and S8). This includes *Laphamia,* whose diversification onset is estimated at 8.7917 Ma [5.3261 to 12.0393 Ma, 95% HPD] yet includes a nested, well-supported clade that contains most desert rock daisies, and whose most recent common ancestor corresponds with the widespread emergence of deserts at 4.9966 Ma [3.0599 to 6.9604 Ma, 95% HPD] (Fig. 2 and *SI Appendix*, Fig. S3*D*).

### Evolution of Life History and Edaphic Specialization onto Bare Rocky Outcrops.

What is the timing and tempo of shifts in life history across the phylogeny of Perityleae? Using reversible jump Markov Chain Monte Carlo (rjMCMC) with stochastic character mapping to infer life-history evolution indicated transition rate asymmetries. Only transitions from suffrutescent perennials to shrubs, perennial herbs, or annuals were inferred as significantly different from zero (95% HPD interval not including 0; 2logBFs > 6; Table 1 and Fig. 3), suggesting homology of the suffrutescent perennial life history across the phylogeny of rock daisies. Model-averaged inference of ancestral states on the maximum a posteriori (MAP) chronogram clarifies that the suffrutescent life history evolved once early in the evolutionary history of rock daisies and is a shared ancestral characteristic of the clade containing *Eutetras*, *Laphamia, Galinsogeopsis,* and *Pericome*, although multiple independent transitions from suffrutescent perennials to shrubs, herbaceous perennials, and annuals occurred more recently within this group (Fig. 3). The most recent common ancestor of the mostly Baja California *Perityle* clade is resolved as an herbaceous perennial, with many descendants of derived annual life history and a woody shrub life history secondarily derived coincident with dispersal to island or island-like habitats in three cases: in *Perityle carterae, Perityle socorrensis,* and *Perityle tenuifolius*.

**Table 1.** Model parameters derived from ancestral state estimates of life history and edaphic affinity during 13,500 post-burn-in iterations of rjMCMC in RevBayes (26).

Character	Transition	Mean rate	Median rate	95% HPD interval	Bayes factor (2logBF)
Life history	Annual to herbaceous perennial	1.7318E-3	1.9846E-4	[0,7.9424E-3]	0.248
	Annual to suffrutescent perennial	1.5406E-3	3.1802E-4	[0,6.735E-3]	0.522
	Annual to shrub	1.651E-3	2.2361E-4	[0,7.1232E-3]	0.280
	Herbaceous perennial to annual	1.5641E-3	1.4279E-4	[0,6.9879E-3]	0.192
	Herbaceous perennial to suffrutescent perennial	1.6333E-3	4.1539E-4	[0,6.8665E-3]	0.605
	Herbaceous perennial to shrub	5.4506E-3	4.6198E-3	[0,0.0133]	5.974
	Suffrutescent perennial to annual	0.0141	0.0132	[4.9633E-3,0.0232]	Inf.
	Suffrutescent perennial to herbaceous perennial	0.0107	0.0102	[3.0716E-3,0.0191]	Inf.
	Suffrutescent perennial to shrub	5.2987E-3	4.8322E-3	[0,0.011]	7.78
	Shrub to annual	1.3456E-3	7.7608E-6	[0,6.1882E-3]	0.016
	Shrub to herbaceous perennial	1.4293E-3	0	[0,6.17151E-3]	0
	Shrub to suffrutescent	1.4289E-3	2.5658E-4	[0,6.1238E-3]	0.377
Edaphic affinity	Habitat generalist to bare rock specialist	4.3876E-3	3.5002E-3	[0,0.035]	Inf.
	Bare rock specialist to habitat generalist	0.0191	0.186	[8.6597E-3,0.0312]	5.766

Bayes factors (BFs) represent support for nonzero transition rates. BFs were calculated as the posterior probability of the nonzero model (the number of MCMC samples that visited the nonzero model divided by the total number of samples) divided by the posterior probability of the zero-rate model (calculated likewise). Equal prior probability was placed on the two models, therefore the prior ratio was not included in the computation of BFs. Following Kass and Raftery (27), 2logBFs were interpreted as: zero to two, equivocal for a nonzero transition rate; two to six, strong support for nonzero transition rate; from six to infinity (inf.), decisive support for a nonzero transition rate.

Is there an evolutionary correlation between life-history evolution and edaphic specialization? Edaphic endemism onto bare rocky outcrops evolved early in the rock daisies and is resolved as the historical ecology of the most recent common ancestor of the clade containing *Eutetras*, *Laphamia, Galinsogeopsis,* and *Pericome.* Growth on decomposed substrates is resolved as the ancestral condition for genus *Perityle* (Fig. 2). RjMCMC-inferred transition rates were not significantly different from zero for transitions onto bare rocky substrates, suggesting that edaphic endemism may have been lost many times, but once lost, was not readily regained (Fig. 3 and Table 1). The timing and tempo of transitions to a suffrutescent life history were mirrored by the evolution of edaphic specialization across rock daisies, hinting at a strong evolutionary dependence between obligate growth on bare rock and the perennating woody caudex that characterizes suffrutescent perennials. Bayesian model testing of dependent [Log-likelihood = −51.090204] and independent [log-likelihood = −65.153536] models of evolution of edaphic endemism and a perennating woody caudex supported this relationship [log Bayes factor = 28.12226], suggesting very strong empirical evidence for evolutionary dependence between these characters across the phylogeny. Transitions to growth on bare rock in plants requires overcoming multiple nonmutually exclusive abiotic and biotic challenges, including lack of soil, increased aridity, exposure to inclement climates, and visibility to natural enemies (28). A perennating woody caudex may serve a dual function under such conditions for anchoring in minute crevices and storing energy during harsh seasons (29); however, prerequisite evolution of such a complex phenotype can also explain why transitions to bare rock have occurred only rarely in the rock daisies.

### Ecological Shifts Inferred with Paleobiome-Informed Models.

What has been the timing and tempo of biome shifts in the rock daisies? Transition rates between desert, tropical, subtropical, and temperate biomes show asymmetries across our study group, with the highest transition rates observed from out-of-tropical and into desert and subtropical biomes, and the lowest a shift from tropical to temperate biomes (Table 2). Rock daisies underwent at least six independent shifts into desert biomes from two geographically separate source areas (Fig. 2). Three of these occurred from out of the Baja California peninsula and three from the Trans-Mexican Volcanic Belt, Sierra Madre Occidental, and Pacific plain of northwest Mexico (Fig. 3). Shifts to desert occurred from tropical habitats in at least four cases and from a subtropical biome in at least one case. Disjunct populations of rock daisies in the Atacama Desert in South America arrived via long-distance dispersal that coincided with a biome shift from tropical environments in southern Baja California and the neighboring Revillagigedo archipelago. The ecological origin of desert dwelling *Galinsogeopsis ciliata* and *G. coronopifolia* was equivocal [ subtropical origin PP = 0.5452, tropical origin PP = 0.4122].

**Table 2.** Results of a biome-shift analysis quantifying relative dependence on three separate underlying q matrices: uninformed (w_u), paleogeographically informed (w_g), and paleobiome-informed (w_b), as well as nonpaleo-biome-informed (w_not_b).

Statistic	Relevance	Mean	Median	95% HPD interval
w_u	Transition matrix uninformed by paleobiome or paleo-geography	0.303	0.277	[0.0146,0.6481]
w_g	Relative importance of paleogeography	0.247	0.282	[4.3735E-4,0.5846]
w_b	Relative dependence on paleobiome structure	0.449	0.45	[0.0419,0.8315]
w_not_b	Independence from paleobiome structure	0.55	0.549	[0.1685,0.9581]

Means, medians, and 95% HPD intervals were estimated using the biome-shift model developed by Landis et al. (15) during 25,000 iterations of MCMC in RevBayes (26). Our analysis considered all combinations of four geographical regions (South America, Baja California, the Trans-Mexican Volcanic Belt, and the Basin and Range Province) with four biomes (tropical, temperate, subtropical, and desert). In addition, our model was structured with four time intervals (Ma): Oligocene (33.9-23.0), early Miocene (23.0-16), mid/late Miocene (16-5.3), and Plio–Pleistocene–Holocene or, here, “recent” (5.3-0). Separation of the Baja California peninsula from mainland Mexico was modeled with weakening connectivity between these areas in the mid/late Miocene and recent time intervals. The recent emergence of widespread arid climates was modeled with increasing connectivity in the recent time interval.

When and from what habitats did rock daisies shift into the NA deserts? The vast majority (~40 spp., >85%) of desert dwelling rock daisies stem from a single shift into the NA deserts from tropical deciduous forests in northwest Mexico shortly after the onset of aridification in the late Miocene (Fig. 2). In contrast, remaining shifts into a desert climate are resolved as recent, occurring along branches with little contemporary lineage diversification. Estimation of transition rates between biomes within a time-integrated model of biome availability and adjacency suggested only moderate dependence of shifts on increasing availability of desert habitats following late Miocene aridification [w_b = 0.45, w_u = 0.303, and w_g = 0.247] (Table 2). Bayesian model testing using rjMCMC further rejects dependence on paleobiome structure [Log Bayes factor > 6] suggesting a model in which shifts into dry habitats were mostly independent from the increase in aridity through time (*SI Appendix*).

### Edaphic Specialization onto Bare Rocky Outcrops as a Precursor to Establishment and Diversification in a Desert Biome.

Did life history evolution associated with edaphic specialization precede establishment of the rock daisies in a NA desert biome? The variable and extreme conditions found in deserts present great challenges for plants, with the lack of water and coarse, rocky soils generally being the most challenging conditions plants face (6–9). These environmentally stressful conditions in desert regions have long been regarded as the stimulus for adaptive strategies for drought tolerance (6, 30). Yet the time-calibrated phylogenetic studies of many desert plant lineages that have been carried out to-date conflict with this conventional view, showing instead that major floristic elements of arid ecosystems worldwide, including cacti, legumes, ocotillos, elephant trees, and others, acquired their unique morphologies prior to the emergence of widespread dry climates in the Neogene and then radiated synchronously during recent onset of aridification (10, 31, *SI Appendix*). This pattern has caused some to speculate that arid biomes appeared much earlier than previously believed, as early as the Eocene (~20 My before the mid-Miocene) (17, 32). How else could these traits have evolved without harsh, arid conditions? We provide support for an alternative hypothesis, that many plants living in a desert environment today did not originally evolve in a desert setting, and that features seen in each plant may not be adaptations to desert living but carryovers from some other set of conditions. Specifically, we find that rock daisies of genus *Laphamia* evolved first as edaphic endemics on “micro deserts” of bare rocky outcrops within otherwise densely vegetated environments, and then spread and diversified more recently under cooler, drier climatic conditions. Most of the ca. 40 minimum-rank taxa of rock daisies in *Laphamia* that currently inhabit the NA deserts show conserved life-history evolution associated with ancestral ecological specialization. Dispersal into the Basin and Range Province when arid conditions intensified in the mid-Miocene evidently led to the ecological release and rapid radiation of *Laphamia* onto ecological islands, including bare, edaphic islands and decomposed substrates, scattered across the varied landscapes of the NA deserts (*SI Appendix*, Fig. S1).

Is edaphic exaptation a common scenario in the evolution of desert biota? The idea that traits conferring drought tolerance may have been a prerequisite rather than a consequence of establishment in the NA deserts is not new (7, 33), yet several nonmutually exclusive lines of evidence suggest it may be a scenario of broader applicability than previously recognized in explaining the enigmatic origins of desert biota. Within forested landscapes, rocky outcrops tend to form microhabitats that consistently harbor a more aridity-resistant flora than their surroundings, and often contain disjunct populations of plants from adjacent, or distant, more open, dry biomes (34, 35). Rocky outcrops in the tropical deciduous forests that are adjacent to the NA deserts, and considered a major source area for expansions into those deserts, harbor familiar members of diverse desert plant and animal groups, including cacti, agaves, euphorbias, rattlesnakes, and lizards (7, *SI Appendix*, Fig. S1). In these and other forested environments, edaphic outcrops have presented a stable and widespread nucleus for the evolution of drought tolerance; in contrast, widespread dry habitats have been rare throughout time, especially during the cool, moist periods of the Oligocene and early Miocene (3, 36, 37).

Traits associated with growth on edaphically harsh substrates overlap with adaptations for drought tolerance. Growth in edaphically arid substrates poses great challenges for plants and many of the adaptive strategies that confer tolerance to stressful soils overlap with those generally considered useful for coping with stress due to aridity (28, 38–40). These traits include perennating organs; dense hairiness; reduction in leaf area; deciduousness; aphylly; tolerance of exposure to UV, intense fluctuations in temperature, wind, and lack of facilitating nurse plants; as well as enhanced herbivore defense strategies to compensate for the high cost of regeneration after attack (28, 38–40). Transitions onto edaphically stressful soils have often been implicated in driving rapid and drastic plant evolution in growth form because of the tradeoffs for resource availability and defense that manifest in nutrient poor conditions (40–43). Tolerance for bare soils has also been implicated in the evolution of further specialization onto chemically unusual serpentine soils, suggesting that bare soils may represent an important hurdle that, once crossed, enables expansions into other stressful niches (40).

What are the forces that maintain edaphic endemism as a stable ecological strategy in dry environments? Edaphic exaptation as a factor in the early evolution of desert angiosperms remains to be reconciled with the theory of fitness trade-offs, which provides a mechanistic explanation for ecological specialization based on free space from competition or natural enemies (42). Most edaphic endemics, including rock daisies, can grow and thrive on zonal (normal) soils in greenhouse conditions, suggesting that tradeoffs in competitive ability or herbivore defense play a crucial role in excluding them from zonal soils in a natural community context (28, 40). In the dense forests where rock daisies evolved edaphic endemism onto bare rocky outcrops, competitive exclusion provides a plausible explanation for the evolution of specialization onto more stressful bare rock. In contemporary deserts, however, the vegetation is open and facilitation by nurse plants is common (9). Escape from competition therefore does not provide a satisfactory mechanism for maintaining soil specialization in deserts. Yet, why do many edaphic specialists occur in arid habitats? One explanation is physiological or ecological constraints associated with past specialization (44). Another is the inverse texture hypothesis (45), which states that in wet ecosystems, where nutrients are more limiting than water, finer, more fertile soils are more productive than coarse, nutrient-poor substrates. However, in dry ecosystems coarser soils allow water to infiltrate below the evaporative zone more rapidly than finer soil, allowing them to retain soil moisture longer. Tests of the inverse soil texture hypothesis are scarce, but, if true, rocky substrates could constitute a crucial biome bridge, facilitating shifts into more arid adaptive zones without fundamental changes to a group’s conserved ecological niche.

## Conclusion

Uncovering the circumstances under which organisms colonized extreme environments such as deserts is an important means of gauging resilience to future environmental stress brought about by climate change. The evolution of drought tolerance in desert plants has often been attributed to adaptation in response to harsh, arid climates. Our phylogenetic analyses of trait evolution and biome-shifts do not support such a model of rapid adaptive evolution in response to dry conditions. In the rock daisies, we find that drought tolerance evolved in association with edaphic specialization onto bare rocky outcrops in a densely vegetated context and was an evolutionary precursor to their extensive diversification in North American deserts. These results add to recent studies highlighting preadaptation (exaptation) and resource tracking as key processes driving innovation and diversification in response to novel selective pressures (37, 38, 46, 47). In addition, this framework suggests that climate change may outpace adaptive evolution in some cases, favoring organismal groups preadapted for stress tolerance to thrive and diversify in novel environments.

## Materials and Methods

### Sampling, Sequencing, Phylogenetic Inference, and Divergence Time Estimation (DTE).

We carried out sequence capture using the COS baits (21, 23, 48) for target capture of ~1,060 low-copy nuclear loci for 114 samples encompassing all minimum-rank taxa of subtribe Peritylinae except for seven taxa for which material was unattainable or sequence yield insufficient, plus three outgroup taxa (*SI Appendix*, Table S1). We extracted DNA, enriched libraries, and processed data using the approach of ref. 21, with the exception that we used iterative reference-based assembly in the HybPiper pipeline (49). We used HybPiper’s paralog investigator script to identify and exclude potentially problematic loci and the intronerate.py script to obtain supercontigs, which included exons and their overlapping flanking introns (see ref. 49 for details). Loci with less than 95% occupancy were removed and the remaining 229 loci aligned using MAFFT (50).

ML and BI trees were generated using RAxML-NG (51) and ExaBayes (52) on the CIPRES science supercomputing portal (53) and a pseudocoalescent tree from gene trees while accounting for ILS was produced using ASTRAL-III (54) (*SI Appendix*, Figs. S2 and S5–S7). A reduced sample dataset for DTE with one representative of each taxon was generated by choosing the sample that conformed most in terms of geography and morphology to the type, retaining individuals of each independent evolutionary lineage when taxa were found to be nonmonophyletic. To this constrained topology, a time-calibrated phylogenetic hypothesis was obtained using Bayesian inference in MCMCtree (55) using a subset of loci and a secondary calibration of the 95% HPD interval estimate of the split of *Helianthus annuus* from Eupatorieae + Perityleae of 7.93 to 23.22 ma, applied to the root of our phylogeny with a uniform prior (24) (*SI Appendix*, Fig. S3).

### Evolution of Life History and Edaphic Specialization.

Observations of life history and edaphic specialization came from herbarium label data, taxonomic treatments, species descriptions, and the 1st author’s fieldtrips to the southwest U.S. and northern Mexico during 2017, 2018, and 2019 (19–21). For the purposes of this study, we considered habit and life history as synonyms. Herbaceous plants included those that have an intraannual or perennial lifecycle and lack woody stem tissue. Suffrutescent perennials resprout annually from a woody stem base, while shrubs are multistemmed and woody above ground. Bare rock surfaces were defined as cliffs, boulders, rocky outcrops, or canyon walls of solid consistency with minimal soil accumulation outside of cracks and crevices. Taxa were considered bare rock specialists if all known occurrences were on solid rock substrates. Taxa were considered habitat generalists if they also, or exclusively, grew on decomposed substrates, which were defined as various weathered soils that fracture on contact and are composed of gravel, sand, silt, or clay. We estimated rates of transitions and inferred ancestral states for life history and edaphic specialization using Bayesian model testing with rjMCMC and stochastic character mapping in RevBayes (26). To test for associations between life history and endemism on rocky outcrops, we used BayesTraits to compare the fit (log-likelihood) of two continuous-time Markov models of life history and edaphic evolution, a dependent and independent model (56).

### Historical Biogeography and Ecology with Paleobiome-Informed Models.

To infer the timing and tempo of biome shifts, we used the biome-shift model developed by Landis et al. (15) as implemented in RevBayes (26) to consider all combinations of four geographical regions (South America, Baja California, the Trans-Mexican Volcanic Belt, and the Basin and Range Province) with four biomes (tropical, temperate, subtropical, and desert). Ancestral biogeographic ranges were additionally inferred and compared with our paleobiome-informed model using a Bayesian implementation of a DEC model in RevBayes (26, 57) (*SI Appendix*, Fig. S4). Our biome-shift model was structured with four time intervals (Ma): Oligocene (33.9-23.0), early Miocene (23.0-16), mid/late Miocene (16-5.3), and Plio–Pleistocene–Holocene or, here, “recent” (5.3-0). South America was coded as having zero connectivity to any other areas in all time intervals. We also modeled the split of the Baja California Peninsula from mainland Mexico beginning ~12 Ma (25) by assigning strong connectivity to the Basin and Range Province and Trans-Mexican Volcanic Belt through the Oligocene and early Miocene, weak connectivity in the mid/late Miocene, and zero connectivity in the recent time interval. Sizes and distributions of temperate, tropical, and subtropical biomes were modeled as constant from the Oligocene to present and desert biomes modeled as emerging with weak connectivity in the late Miocene and strong connectivity in the recent time interval.

Additional details of the methods are available in *SI Appendix*.

## Supplementary Material

Appendix 01 (PDF)

## Data Availability

Raw sequencing reads were deposited in the National Center for Biotechnology Information (NCBI) Sequence Read Archive under BioProject PRJNA918694 (58).

## References

[1] F. T. Maestre , Biogeography of global drylands. New Phytol. **231**, 540–558 (2021). 33864276 10.1111/nph.17395

[2] J. T. Overpeck, B. Udall, Climate change and the aridification of North America. Proc. Natl. Acad. Sci. U.S.A. **117**, 11856–11858 (2020). 32430321 10.1073/pnas.2006323117 PMC7275756

[3] A. Graham, The age and diversification of terrestrial New World ecosystems through Cretaceous and Cenozoic time. Am. J. Bot. **98**, 336–351 (2011). 21613130 10.3732/ajb.1000353

[4] F. Shreve, I. L. Wiggins, Vegetation and Flora of the Sonoran Desert (Stanford University Press, 1964).

[5] T. D. Herbert , Late Miocene global cooling and the rise of modern ecosystems. Nat. Geosci. **9**, 843–847 (2016).

[6] G. L. Stebbins Jr., Aridity as a stimulus to plant evolution. Am. Nat. **86**, 33–44 (1952).

[7] D. I. Axelrod, Edaphic aridity as a factor in angiosperm evolution. Am. Nat. **106**, 311–320 (1972).

[8] S. D. Smith, R. Monson, J. E. Anderson, Physiological Ecology of North American Desert Plants (Springer Science & Business Media, 2012).

[9] J. Belnap , “Deserts” in Ecosystems of California, H. Mooney, E. Zavaleta, Eds. (University of California Press, 2016), pp. 635–669.

[10] M. Arakaki , Contemporaneous and recent radiations of the world’s major succulent plant lineages. Proc. Natl. Acad. Sci. U.S.A. **108**, 8379–8384 (2011). 21536881 10.1073/pnas.1100628108 PMC3100969

[11] M. J. Moore, R. K. Jansen, Molecular evidence for the age, origin, and evolutionary history of the American desert plant genus *Tiquilia* (Boraginaceae). Mol. Phylogenet. Evol. **39**, 668–687 (2006). 16495087 10.1016/j.ympev.2006.01.020

[12] S. V. Good-Avila, V. Souza, B. S. Gaut, L. E. Eguiarte, Timing and rate of speciation in *Agave* (Agavaceae). Proc. Natl. Acad. Sci. U.S.A. **103**, 9124–9129 (2006). 16757559 10.1073/pnas.0603312103 PMC1482577

[13] M. J. Donoghue, A phylogenetic perspective on the distribution of plant diversity. Proc. Natl. Acad. Sci. U.S.A. **105**, 11549–11555 (2008). 18695216 10.1073/pnas.0801962105 PMC2556411

[14] M. J. Donoghue, E. J. Edwards, Biome shifts and niche evolution in plants. Annu. Rev. Ecol. Evol. Syst. **45**, 547–572 (2014).

[15] M. Landis, E. J. Edwards, M. J. Donoghue, Modeling phylogenetic biome shifts on a planet with a past. Syst. Biol. **70**, 86–107 (2021). 32514540 10.1093/sysbio/syaa045

[16] L. Palazzesi, O. Hidalgo, V. D. Barreda, F. Forest, S. Höhna, The rise of grasslands is linked to atmospheric CO_2_ decline in the late Palaeogene. Nat. Commun. **13**, 1–10 (2022). 35022396 10.1038/s41467-021-27897-y PMC8755714

[17] E. Gagnon, J. J. Ringelberg, A. Bruneau, G. P. Lewis, C. E. Hughes, Global succulent biome phylogenetic conservatism across the pantropical Caesalpinia Group (Leguminosae). New Phytol. **222**, 1994–2008 (2019). 30536385 10.1111/nph.15633

[18] P. A. Christin , Anatomical enablers and the evolution of C_4_ photosynthesis in grasses. Proc. Natl. Acad. Sci. U.S.A. **110**, 1381–1386 (2013). 23267116 10.1073/pnas.1216777110 PMC3557070

[19] A. M. Powell, Taxonomy of *Perityle* section *Perityle* (Compositae—Peritylinae). Rhodora **76**, 229–306 (1974).

[20] I. H. Lichter-Marck, B. G. Baldwin, A phylogenetically informed reclassification of Perityleae. Syst. Bot. **47**, 802–816 (2022).

[21] I. H. Lichter-Marck , Phylogenomics of Perityleae (Compositae) provides new insights into morphological and chromosomal evolution of the rock daisies. J. Syst. Evol. **58**, 853–880 (2020).

[22] NatureServe, NatureServe Network Biodiversity Location Data. Available at www.natureserve.com. Accessed 15 October 2022.

[23] J. R. Mandel , A fully resolved backbone phylogeny reveals numerous dispersals and explosive diversifications throughout the history of Asteraceae. Proc. Natl. Acad. Sci. U.S.A. **116**, 14083–14088 (2019). 31209018 10.1073/pnas.1903871116 PMC6628808

[24] M. J. Landis, W. A. Freyman, B. G. Baldwin, Retracing the Hawaiian silversword radiation despite phylogenetic, biogeographic, and paleogeographic uncertainty. Evolution **72**, 2343–2359 (2018). 30198108 10.1111/evo.13594

[25] T. R. Van Devender, “Deep history and biogeography of la frontera” in Changing Plant Life of La Frontera: Observations on Vegetation in the United States/Mexico Borderlands, G.L. Webster, C. J. Bahre, Eds. (UNM Press, 2001), pp. 121–134.

[26] S. Höhna, M. J. Landis, RevBayes: Bayesian phylogenetic inference using graphical models and an interactive model-specification language. Syst. Biol. **65**, 726–736 (2016). 27235697 10.1093/sysbio/syw021 PMC4911942

[27] R. E. Kass, A. E. Raftery, Bayes factors. J. Am. Stat. Assoc. **90**, 773–795 (1995).

[28] N. I. Cacho, P. J. McIntyre, “The role of enemies in bare and edaphically challenging environments” in Evolutionary Ecology of Plant-Herbivore Interaction, J. Núñez-Farfán, P. Valverde, Eds. (Springer, 2020), pp. 249–267.

[29] P. Poot, S. D. Hopper, J. M. van Diggelen, Exploring rock fissures: Does a specialized root morphology explain endemism on granite outcrops? Ann. Bot. **110**, 291–300 (2012). 22238122 10.1093/aob/mcr322 PMC3394634

[30] D. T. MacDougal, Influence of aridity upon the evolutionary development of plants. Plant World **12**, 217–231 (1909).

[31] A. Weeks , To move or to evolve: Contrasting patterns of intercontinental connectivity and climatic niche evolution in “Terebinthaceae” (Anacardiaceae and Burseraceae). Front. Genet. **5**, 409 (2014). 25506354 10.3389/fgene.2014.00409 PMC4247111

[32] B. D. Schrire, M. T. Lavin, G. P. Lewis, “Global distribution patterns of the Leguminosae: Insights from recent phylogenies” In Plant Diversity and Complexity Patterns: Local, Regional and Global Dimensions. Proceedings of an International Symposium held at the Royal Danish Academy of Sciences and Letters in Copenhagen, Denmark, 25-28 May, 2003 (Det Kongelige Danske Videnskabernes Selskab, 2003), pp. 375–422.

[33] D. I. Axelrod, Evolution of the Madro-Tertiary geoflora. Bot. Rev. **24**, 433–509 (1958).

[34] S. D. Wesser, W. S. Armbruster, Species distribution controls across a forest-steppe transition: A causal model and experimental test. Ecol. Monogr. **61**, 323–342 (1991).

[35] Z. Bátori , Karst dolines provide diverse microhabitats for different functional groups in multiple phyla. Sci. Rep. **9**, 1–13 (2019). 31073136 10.1038/s41598-019-43603-x PMC6509348

[36] I. Agarwal, T. Thackeray, S. Pal, A. Khandekar, Granite boulders act as deep-time climate refugia: A Miocene divergent clade of rupicolous *Cnemaspis* Strauch, 1887 (Squamata: Gekkonidae) from the Mysore Plateau, India, with descriptions of three new species. J. Zool. Syst. Evol. Res. **58**, 1234–1261 (2020).

[37] R. T. Corlett, K. W. Tomlinson, Climate change and edaphic specialists: Irresistible force meets immovable object? Trends Ecol. Evol. **35**, 367–376 (2020). 31959419 10.1016/j.tree.2019.12.007

[38] J. A. Fitzsimons, D. R. Michael, Rocky outcrops: A hard road in the conservation of critical habitats. Biol. Conserv. **211**, 36–44 (2017).

[39] K. U. Brady, A. R. Kruckeberg, H. D. Bradshaw Jr., Evolutionary ecology of plant adaptation to serpentine soils. Annu. Rev. Ecol. Evol. Syst. **36**, 243–266 (2005).

[40] N. I. Cacho, S. Y. Strauss, Occupation of bare habitats, an evolutionary precursor to soil specialization in plants. Proc. Natl. Acad. Sci. U.S.A. **111**, 15132–15137 (2014). 25267640 10.1073/pnas.1409242111 PMC4210328

[41] R. A. Folk, C. M. Siniscalchi, D. E. Soltis, Angiosperms at the edge: Extremity, diversity, and phylogeny. Plant Cell Environ. **43**, 2871–2893 (2020). 32926444 10.1111/pce.13887

[42] P. A. Fine, D. C. Daly, K. M. Cameron, The contribution of edaphic heterogeneity to the evolution and diversity of Burseraceae trees in the western Amazon. Evolution **59**, 1464–1478 (2005). 16153032

[43] D. I. Axelrod, Drought, diastrophism, and quantum evolution. Evolution **21**, 201–209 (1967). 28556140 10.1111/j.1558-5646.1967.tb00149.x

[44] A. Vicentini, The evolutionary history of *Pagamea* (Rubiaceae), a white-sand specialist lineage in tropical South America. Biotropica **48**, 58–69 (2016).

[45] O. E. Sala, W. J. Parton, L. A. Joyce, W. K. Lauenroth, Primary production of the central grassland region of the United States. Ecology **69**, 40–45 (1988).

[46] M. J. Endara , Coevolutionary arms race versus host defense chase in a tropical herbivore–plant system. Proc. Natl. Acad. Sci. U.S.A. **114**, 7499–7505 (2017). 10.1073/pnas.1707727114 PMC5594685 28827317

[47] W. S. Armbruster, J. Lee, B. G. Baldwin, Macroevolutionary patterns of defense and pollination in *Dalechampia* vines: Adaptation, exaptation, and evolutionary novelty. Proc. Natl. Acad. Sci. U.S.A. **106**, 18085–18090 (2009). 19841278 10.1073/pnas.0907051106 PMC2775306

[48] J. R. Mandel , A target enrichment method for gathering phylogenetic information from hundreds of loci: An example from the Compositae. Appl. Plant Sci. **2**, 1300085 (2014). 10.3732/apps.1300085 PMC4103609 25202605

[49] M. G. Johnson , HybPiper: Extracting coding sequence and introns for phylogenetics from high-throughput sequencing reads using target enrichment. Appl. Plant Sci. **4**, 1600016 (2016). 10.3732/apps.1600016 PMC4948903 27437175

[50] K. Katoh, G. Asimenos, H. Toh, “Multiple alignment of DNA sequences with MAFFT” in Bioinformatics for DNA Sequence Analysis, D. Posada, Ed. (Humana Press, 2009), pp. 39–64. 10.1007/978-1-59745-251-9_3 19378139

[51] A. M. Kozlov, D. Darriba, T. Flouri, B. Morel, A. Stamatakis, RAxML-NG: A fast, scalable and user-friendly tool for maximum likelihood phylogenetic inference. Bioinformatics **35**, 4453–4455 (2019). 31070718 10.1093/bioinformatics/btz305 PMC6821337

[52] A. J. Aberer, K. Kobert, A. Stamatakis, ExaBayes: Massively parallel Bayesian tree inference for the whole-genome era. Mol. Biol. Evol. **31**, 2553–2556 (2014). 25135941 10.1093/molbev/msu236 PMC4166930

[53] M. A. Miller , A RESTful API for access to phylogenetic tools via the CIPRES science gateway. Evol. Bioinform. **11**, EBO-S21501 (2015). 10.4137/EBO.S21501 PMC4362911 25861210

[54] C. Zhang, E. Sayyari, S. Mirarab, “ASTRAL-III: Increased scalability and impacts of contracting low support branches” in RECOMB International Workshop on Comparative Genomics (Springer, 2017), pp. 53–75.

[55] M. Dos Reis, P. C. Donoghue, Z. Yang, Bayesian molecular clock dating of species divergences in the genomics era. Nat. Rev. Genet **17**, 71–80 (2016). 26688196 10.1038/nrg.2015.8

[56] M. Pagel, A. Meade, Bayesian analysis of correlated evolution of discrete characters by reversible-jump Markov chain Monte Carlo. Am. Nat. **167**, 808–825 (2006). 16685633 10.1086/503444

[57] R. H. Ree, S. A. Smith, Maximum likelihood inference of geographic range evolution by dispersal, local extinction, and cladogenesis. Syst. Biol. **57**, 4–14 (2008). 18253896 10.1080/10635150701883881

[58] IH Lichter-Marck, BioProject PRJNA918694: Edaphic specialization onto bare, rocky outcrops as a factor in the evolution of desert angiosperms, National Center for Biotechnology Information Sequence Read Archive/ **57**, 4–14 (2008). www.ncbi.nlm.nih.gov/bioproject/PRJNA918694, January 5th 2023. 10.1073/pnas.2214729120 PMC9963280 36716359

